# Role of Interleukin-4 (IL-4) in Respiratory Infection and Allergy Caused by Early-Life *Chlamydia* Infection

**DOI:** 10.4014/jmb.2104.04028

**Published:** 2021-06-29

**Authors:** Shujun Li, Lijuan Wang, Yulong Zhang, Long Ma, Jing Zhang, Jianbing Zu, Xuecheng Wu

**Affiliations:** 1 Department of Clinical Laboratory, People's Hospital of Xing'an League, Ulanhot, Inner Mongolia 137400, P.R. China; 2 Department of Five Sense Organs, People's Hospital of Xing'an League, Ulanhot, Inner Mongolia 137400, P.R. China; 3 Department of Clinical Laboratory, Shenzhen People's Hospital, Shenzhen, Guangdong 518110, P.R. China

**Keywords:** *Chlamydia*, respiratory infection, interleukin-4, asthma, pneumonia

## Abstract

*Chlamydia pneumoniae* is a type of pathogenic gram-negative bacteria that causes various respiratory tract infections including asthma. *Chlamydia* species infect humans and cause respiratory infection by rupturing the lining of the respiratory which includes the throat, lungs and windpipe. Meanwhile, the function of interleukin-4 (IL-4) in *Ch. pneumoniae* respiratory infection and its association with the development of airway hyperresponsiveness (AHR) in adulthood and causing allergic airway disease (AAD) are not understood properly. We therefore investigated the role of IL-4 in respiratory infection and allergy caused by early life *Chlamydia* infection. In this study, *Ch. pneumonia* strain was propagated and cultured in HEp-2 cells according to standard protocol and infant C57BL/6 mice around 3-4 weeks old were infected to study the role of IL-4 in respiratory infection and allergy caused by early life *Chlamydia* infection. We observed that IL-4 is linked with *Chlamydia* respiratory infection and its absence lowers respiratory infection. IL-4R α2 is also responsible for controlling the IL-4 signaling pathway and averts the progression of infection and inflammation. Furthermore, the IL-4 signaling pathway also influences infection-induced AHR and aids in increasing AAD severity. STAT6 also promotes respiratory infection caused by *Ch. pneumoniae* and further enhanced its downstream process. Our study concluded that IL-4 is a potential target for preventing infection-induced AHR and severe asthma.

## Introduction

*Chlamydia* (Ch) species are a type of pathogenic gram-negative bacteria that cause various respiratory tract infections including pharyngitis, bronchitis, and pneumonia [1]. They are obligate intracellular parasites and their infection also causes bacterial sexually transmitted diseases among humans [2]. The most common species of *Chlamydia* are *Ch. pneumonia*, *Ch. abortus*, *Ch. psittaci*, *Ch. trachomatis* (which infects humans), *Ch. muridarum* (which only infects rodents and mice) and *Ch. suis* (which infects swine) [3]. The species infect humans and cause respiratory infection by rupturing the lining of the respiratory tract including the throat, lungs and windpipe [4]. Most people get infected with *Ch. pneumoniae* and may have mild symptoms or no symptoms at all [5]. In most cases Ch. Pneumonia infections occur for the first time during school days or young age [6]. The common symptoms of this bacterial infection are sore throat, runny nose, fatigue, mild fever, and headache [7]. Respiratory infections caused by *Ch. pneumoniae* are common during early life and many teenagers who showed asymptomatic symptoms are the root cause of of community-acquired pneumonia, particularly among the newborn and pediatric population [8]. On the other hand, the cytokine interleukin 4 (IL-4) plays a major role in the induction and differentiation of naive helper T- helper cells from Th0 to Th2 cells. IL-4 is mainly produced by mast cells, basophils, and eosinophils, and when it is activated, the Th2 cell generates extra IL-4 [9]. The function of IL-4 is quite similar to that of IL-13 and both of them are closely associated. IL-4 is known to play a crucial role the pathogenesis of asthma and other inflammatory responses arising from respiratory infection. There are also reports which link the association of *Ch. pneumoniae* infections in the lungs with the onset of asthma and wheezing [10]. One of the underlying causes of asthma and inflammatory responses of respiratory infection are induced by Th-2-type cytokines such as IL-4 and IL-13 [11]. These cytokines alerts the IL-4Rα/IL-13Rα1 receptor of the signal transducer and activator of transcription 6 (STAT6) thereby promoting inflammation, mucus-secreting cell (MSC) metaplasia/hyperplasia, and airway hyper responsiveness (AHR). In spite of these reports, the underlying mechanism of IL-4 is not properly understood with respect to respiratory infection [12, 13]. It also accumulates the expression of alternatively activated macrophage (AAM) genes and this phenotype differentiates in response to IL-4/IL-13-mediated activation of STAT6, thus possessing potent inhibitory activity against T cells [14]. It also plays a major role in the induction and progression of various respiratory infection diseases. There are reports on the constriction of bronchitis due to heightened AHR which leads to breathing difficulties and wheezing [15]. There are also reports that *Chlamydia* infection in early life reduces the activity of the lung and diminishes its function in the long run by causing AHR during adulthood and leading to allergic airway disease (AAD) [16]. The function of IL-4 in *Ch. pneumoniae* respiratory infection during early life and its association with AHR and AAD development require further analysis. Therefore, the present study is an attempt to study the role of IL-4 in respiratory infection and allergy caused by early-life *Ch. pneumoniae* infection using a mouse model.

## Materials and Methods

### Ethics Approval and Consent to Participate

All animal experiments were performed according to the guidelines and protocols approved by the Institutional Research Review and Ethics Board of Shenzhen Peoplés Hospital, Shenzhen, Guangdong, China, 518110 vide approval No. S21-21565 of Project-81900231. All experiments were designed in accordance with the Guide for the Care and Use of Laboratory Animals published by the National Institutes of Health (NIH Pub. No. 85-23, revised 1996). All experiments were carried out in strict accordance with the recommendations according to international practices for the care and use of animals for scientific purposes.

### Mice

Infant C57BL/6 mice around 3-4 weeks old were obtained from the institute lab and used throughout the study. The mice were caged under specific pathogen-free condition.

### *Ch. pneumoniae* Infection

*Ch. pneumoniae* strain was propagated and cultured in HEp-2 cells according to the standard protocol. The cells were checked for mycoplasma contamination using PCR and the Inclusion-Forming Units (IFUs) were assessed by suspension in a 0.2 M sucrose, 0.02 M sodium phosphate (pH 7.2), 5 mM glutamate buffer. The mice were then sacrificed at 10, 20, 30, and 40 dpi (days post infection) to assess infection and pathology. Lungs were collected at various time points after infection and used for analysis of cell numbers.

### Flow Cytometry

The cell suspensions of the digested lungs were stimulated with 01. μg/ml of PMA and stained with their specific surface marker. Then, they were fixed with 4 % paraformaldehyde and permeabilized. The cell populations were analyzed using an Attune NxT Flow Cytometer (Thermo Fisher Scientific China Co., Ltd.)

### Nucleic Acid and Protein Isolations

The nucleic acid and protein present in the lung homogenates were extracted using Standard Isolation and Protein Purification kits (Thermo Fisher). The *Chlamydia* numbers were quantified using real-time quantitative PCR based on standard protocol.

### Histopathology Analysis

The lung sections were fixed with formalin and stained with hematoxylin and eosin and sectioned with a thickness of 0.5 microns. The stained sections and MSC numbers were quantified and scored by the pathologist.

### RNA Extraction and qRT-PCR

RNA was extracted from the homogenates of the lungs using Standard Kit (Invitrogen, China) following the manufacturer’s protocol. The extracted RNA was further treated with DNAsel and reverse transcribed using specific primers. Quick RT-PCR was used to evaluate the relative abundance of cDNA and compared using the reference gene hypoxanthine-guanine phosphoribosyltransferase (HPRT) and the mRNA expression of IL-4 was determined.

### ELISA Assay for Detection of Specific Antibodies

Total protein from the homogenates of lung were assayed using BCA assay and the concentrations of IL-4 and IL4Rα2 lung homogenates were determined using ELISA assay. *Ch. pneumoniae* were cultured in FCS containing 1.2 mml/l of HEPES solution supplemented with an antibiotic. The culture supernatants were evaluated using an ELISA plate reader.

### Lung Function

The AHR analysis for lung function was evaluated by anesthetizing the mice. The peak of the transpulmonary resistance and dynamic compliance were measured with the increasing dose of methacholine.

### Statistical Analysis

Statistical analysis was carried out using SPSS 18.0 (SPSS Inc., USA). All results are expressed as mean ± SE. Multiple comparisons for statistical significance were calculated using two-way ANOVA. *p*-value < 5.0 was considered statistically significant.

## Results

### IL-4 is Linked with *Chlamydia* Respiratory Infection and Its Absence Lowers Respiratory Infection

**Fig. 1:**
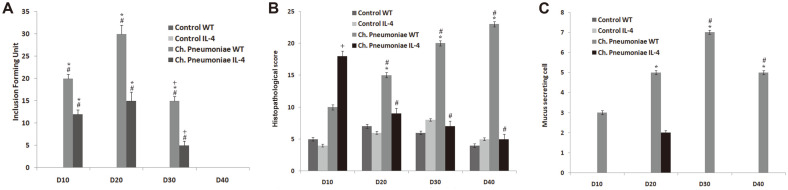
*Ch. pneumoniae* respiratory infection in early life and infection-induced histopathological results. (**A**) Quantification of lung homogenates during *Ch. pneumonia* load, (**B**) histopathological score of lung tissue and (**C**) cell count of mucus-secreting cells. All results are expressed as mean ± SE. * and + corresponds to *p* < 0.05 compared with Control WT and Control IL4. # corresponds to *p* < 0.05 compared with *Ch. pneumoniae* WT.

**Fig. 2:**
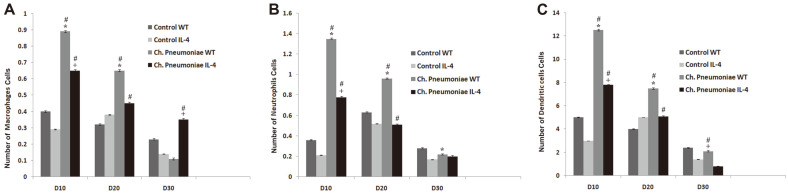
*Ch. pneumoniae* respiratory infection-induced pulmonary inflammation. Flow cytometry results showing the number of inflammatory cells for (**A**) macrophages (**B**) neutrophils and (**C**) dendritic cells. All results are expressed as mean ± SE. * and + corresponds to *p* ≤ 0.05 compared with Control WT and Control IL4. # corresponds to *p* ≤ 0.05compared with *Ch. pneumoniae* WT.

The assessment analysis from the infection of *Ch. pneumoniae* in the lungs of the 3-week-old wild-type and IL4-/- mice observed that there was a considerable load at 10 dpi and peak at 20 dpi whereas the load decreases at 30 dpi and cleared totally at 40 dpi. However, in case of IL4-/- mice, the load was observed between 10 to 20 dpi and there was a considerable reduction between 30 to 40 dpi and the infection was totally cleared by 40 dpi. Interestingly, the IL4-/- mice were able to reduce the *Ch. pnumoniae* load at 10, 20, 30, and 40 dpi when compared to the infected WT controls (Fig. 1). The histopathology analysis of IL4-/- mice also had a reduced mark of pulmonary inflammation (Fig. 1B). In Fig. 1 it is also observed that the absence of IL-4 reduces *Chlamydia* respiratory infection in early life and infection-induced histopathology. The results are expressed as mean ±SE with *p* < 0.05 compared with Control WT and Control IL4. The influx of the inflammatory cells also peaked with increasing numbers of macrophages, neutrophils, and dendritic cells which suggested that the absence of IL-4 reduces infection-induced pulmonary inflammation (Fig. 2). Results are expressed as mean ± SE with *p* ≤ 0.05 compared with Control WT and Control IL4.

### Association of IL4 Level and Early-Life *Ch. pneumonia* Infection

**Fig. 3:**
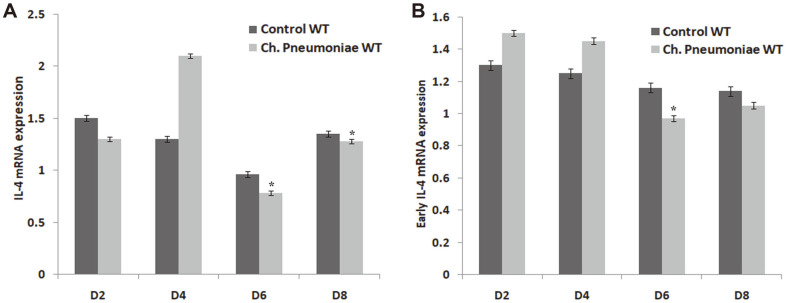
Assessment analysis of (**A**) IL-4 mRNA expression and (**B**) early IL-4 mRNA expression from the lung homogenates using RT-PCR and ELISA assay. Results are expressed as mean ± SE. *corresponds to *p* ≤ 0.05 compared with Control WT.

The study also observed that IL-4-/- infected mice showed drastic reduction in *Chlamydia* load after the infection with *Ch. pneumonia*. The result of the infection affecting the level of IL-4 in WT mice is presented in Figs. 3A and 3B. However, the study also showed that the mRNA expression and protein level of IL-4 in lung homogenates did not increase during the course of infection compared to the Control WT mice, thus indicating that *Ch. pneumoniae* infection during early life does not increase the levels of IL-4. Moreover, the rate of infection was unable to induce the IL-4 response without proper expression of IL-4 mRNA or protein level (Fig. 3).

### Effect of IL-4 mRNA Expression

**Fig. 4:**
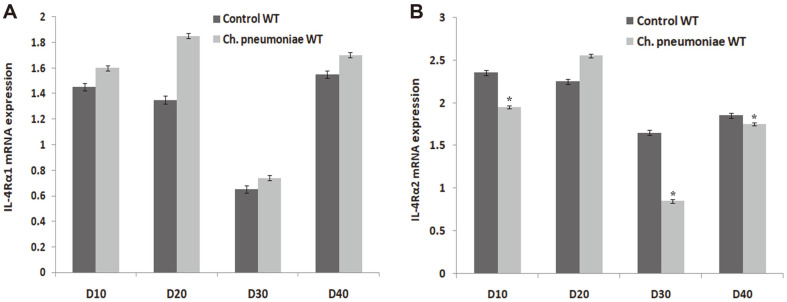
ELISA assay expression and protein production of IL-4 showing the receptor levels of (**A**) IL-4α1 and (**B**) IL-4α2 in lung homogenates assessed by qPCR and ELISA assay. Results are expressed as mean ± SE. *corresponds to *p* ≤ 0.05 compared with Control WT.

The evaluation on the effect of IL-4 receptor level in order to confirm the effect of IL-4 mRNA expression revealed that there was no major variation in the homogenates compared to Control WT as shown in Figs. 4A and 4B. This result showed that *Ch. pneumoniae* infection during early life reduces the production of IL-4 decoy receptor. However, the expression level of both IL-4R α2 mRNA and IL-4R α2 protein expression decreased at 30 dpi with a *p* ≤ 0.05 compared with Control WT (Fig. 4).

### Association of AAM Gene Expression and IL-4 in *Chlamydia*-Infected Lungs

**Fig. 5:**
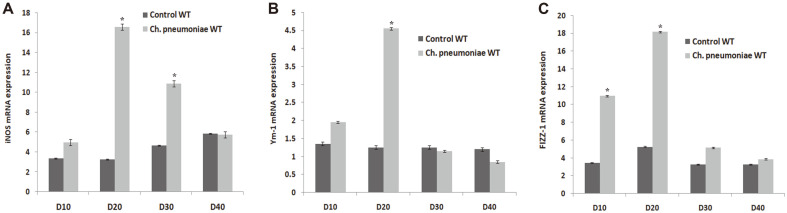
Expression of alternatively activated macrophage genes by *Ch. pneumoniae* infection representing (**A**) iNOS (**B**) Ym-1 and (**C**) FIZZ-1 assessed by qPCR. Results are expressed as mean ± SE. *corresponds to *p* ≤ 0.05 compared with Control WT.

The effect of *Chlamydia* infection on the expression of AAM gene observed that the expression level of iNOS, Ym-1, and FIZZ-1 mRNA of *Ch. pneumoniae*-infected WT mice in the lung homogenates was significantly high compared to the Control WT (Fig. 5). The factors for the gene expression were mostly stimulated at 10 dpi in the WT mice and there was an increased level of iNOS, Arg-1, Ym-1 and FIZZ-1 in the IL-4−/− mice compared to Control WT (Fig. 5). Thus, it is revealed that IL-4 promotes the expression of AAM gene in the early-life infection.

## Discussion

The present study showed that *Chlamydia* infection causes respiratory distress in early life and suppresses the IL-4 alpha thereby enabling the IL-4 to promote infection. This leads to an infection-induced AHR and results in increased severity of AAD in future days. In fact, IL-4 is produced primarily by Th2 cells upon *Chlamydia* infection in adults. It further activated the mast cells and eosinophils which is required for Th2 cell differentiation and expansion [17]. In adults, *Chlamydia* infections typically induce potent and protective T helper cells. But in early life, the role of Th2 cells in *Chlamydia* infection is not well studied and understood properly. This is because, in early life, the factors induced by *Chlamydia* infection which are involved in clearance are not well studied and difficult to trace [18]. Chlamydial infections (RTIs) are usually considered as an example of how infections in early life promote the developmental origins of disease and asthma [19]. Moreover, 80% of adults have anti-chlamydial antibodies, suggesting that these individuals have been infected at some stage of their lives [20]. Moreover, during *Chlamydia* infection, IL-4 is necessary for Th2 cell differentiation. It also promotes the level of immunoglobulin E (IgE) production, allergic inflammation and the development of mucus hypersecretion and AHR. [21]. IL4 also promotes isotype switching of B cells to IgE production, thereby leading to the growth and development of mast cells and eosinophils [22, 23]. IL-4 also contributed in maintaining the inflammatory response to antigens, the production of eotaxins and the development of mucus-secreting cells and airway hyper responsiveness [24, 25]. In the present study, it is observed that the level of IL-4Rα2 is suppressed by *Ch. pneumoniae* respiratory infection which enables the IL-4 in promoting further infection and severity of AAD. The study demonstrates the infection of *Ch. pneumoniae* using mouse model which creates and induces various immune responses and pathological features which are also observed in humans and includes mucus hyper secretion, cell inflammation and damaged lung [26]. Another interesting observation is that the severity of infection and histopathology influenced by infection was significantly reduced in the absence of IL-4. It is also associated with reduced number of inflammatory cells which get into the lungs [27]. However, the expression level of IL-4 is not influenced by early-life *Ch. pneumoniae* infection but it diminishes the level of IL-4Rα2, thereby taking out the major regulatory mechanisms that control the IL-4 signaling pathway [28]. *Ch. pneumoniae* infection in mice also leads to the development of AAM phenotype but the same was decreased in IL-4 -/- mice. Additionally, *Ch. pneumoniae* infection in WT mice during early life were able to induce AHR development which continued till their adulthood. In case of infected WT mice, the injection of IL-4 to IL-4-/- mice restored their phenotype. However, the offset of IL-4 infection in early life intercepts the process of AHR development and AAD severity [29]. Earlier, it was reported that the infection of *Chlamydia* leads to rapid production of IL-4/ IL-13. Their study also observed that there was an increased uptake of macrophage and reduced level of IL-4/ IL-13 [30]. An interesting observation in their study is that the absence of IL-4/ IL-13 expression during their early-life Ch. pnenumoniae infection, the severity, mucus hypersecretion and inflammation of infection was comparatively low. The present study also observed that the *Ch. pneumoniae* infection during early life reduced the expression level of IL-4R. Therefore, it allows the constitutive IL-4 to induce with more effects and activates the STAT6 signaling pathway which leads to MSC hyperplasia and inflammation [31]. The study demonstrates that *Ch. pneumoniae* infection reduces the level of IL-4Rα2 present in bronchoalveolar and blood serum. However, the amount of soluble IL-4Rα2 was not present in blood serum or bronchoalveolar of the control subjects. In addition, it is reported that soluble IL-4Rα2 acts as an important indicator for the activity of IL-4 regulation in human lung tissues. Their study further implies that the enzymatic activity of IL-4Rα2 allowed them to cleave from its main surface for generation of its soluble counterpart [32]. Hence, the presence of soluble IL-4Rα2 in the lungs of human is considered as a major process during *Chlamydia* infection and other associated diseases such as asthma [33]. Further, it is confirmed that the persistence of AHR development is induced by *Chlamydia* infection during early life and not during adulthood.

In our study, the mechanisms involved during early-life *Ch. pneumoniae* infection showed that the IL-4 expression during their early-life infection enhanced the development of AHR thereby suppressing the production of IL-4Rα2. IL-4 also activates the STAT6 signaling pathway through the tyrosine phosphorylation and this downstream process of IL-4 has been observed to aid in IL-4 inducing effects such as inflammation, gene expression of AHR and AAM [21]. In our study it was observed that the STAT6 signaling pathway played a significant role in *Ch. pneumonaie* infection. In the absence of STAT6, the *Ch. pneumonia* infected AAM gene expression including the *Chlamydia* load, and AHR was totally suppressed. However, STAT6-/- mice were vulnerable to *Chlamydia* infection, thus revealing that IL-4-mediated STAT6 signaling pathway triggered and induced the infection. The study observed that IL-4R α2 is responsible for controlling the IL-4 signaling pathway in early-life *Ch. pneumonaie* infection. It also averted the progression of infection and inflammation. The IL-4 signaling pathway also influences the infection-induced AHR and increases AAD severity. In addition, it was observed that STAT6 also promotes respiratory infection caused by *Ch. pneumoniae* and further enhanced the downstream process. Therefore, the study concludes that IL-4 and STAT6 are potential targets for preventing infection-induced AHR and severity of asthma in future life.
